# Impact of Residual Vein Venous Thrombosis in Consecutive Patients with Cancer-Associated Thrombosis Treated with Tinzaparin—A Cohort Study

**DOI:** 10.3390/cancers16213591

**Published:** 2024-10-24

**Authors:** Carmen Rosa-Linares, Maria Barca-Hernando, Victor Garcia-Garcia, Sergio Lopez-Ruz, Teresa Elias-Hernandez, Remedios Otero-Candelera, David Gutierrez-Campos, Henry Andrade-Ruiz, Luis Jara-Palomares

**Affiliations:** 1 Respiratory Department, Medical Surgical Unit of Respiratory Diseases, Hospital Virgen del Rocio, 41013 Seville, Spain; carmen.rosa.linares.sspa@juntadeandalucia.es (C.R.-L.); maria.barca.sspa@juntadeandalucia.es (M.B.-H.); victor.garcia.garcia.sspa@juntadeandalucia.es (V.G.-G.); sergio.lopez.sspa@juntadeandalucia.es (S.L.-R.); teresa.elias.sspa@juntadeandalucia.es (T.E.-H.); rotero@separ.es (R.O.-C.); dantonio.gutierrez@juntadeandalucia.es (D.G.-C.); 2 Center for Biomedical Research in the Respiratory Diseases Network (CIBERES), Instituto de Salud Carlos III, 28029 Madrid, Spain; 3 Foundation for Health Research Management—FISEVI, 41013 Seville, Spain; hantonio.andrade@juntadeandalucia.es

**Keywords:** cancer, metastatic cancer, venous thromboembolism, pulmonary embolism, residual venous thrombosis

## Abstract

**Background**: The role of residual venous thrombosis (RVT) as a risk factor for recurrent venous thromboembolism (VTE) in patients with cancer-associated thrombosis (CAT) remains controversial. **Methods**: We conducted a cohort study on consecutive patients with CAT treated with tinzaparin recruited between 2007 and 2022. Primary outcome: RVT. Secondary outcomes: identification of variables associated with RVT and the role of RVT in VTE recurrences or clinically relevant bleeding (CRB). **Results**: Among 511 patients with CAT (age 64.1 years ± 13.4 years; 53.5% males) followed for 17.6 months (p25–75: 7.9–34), 35.8% (n = 183) presented RVT (at 6 months, 55.5%). Variables identified as being associated with RVT were ECOG performance status > 1, metastasis, and cancer location. Within 5 years, there were 57 CRB (11.2%; 95% CI: 8.6–14.2) and 67 VTE recurrences (13.1%, 95%CI: 10.3–16.4). Competing risk analysis identified that RVT at 6 months was associated with VTE recurrence within 5 years (sub-hazard ratio: 2.1; 95% CI: 1.2–3.7; *p* = 0.006), but not with CRB. Multivariate analysis confirmed that RVT at 6 months (HR: 2.1; 95% CI: 1.2–3.7) and metastases (HR: 1.7; 95% CI: 1.1–2.9) were associated with VTE recurrence within 5 years. **Conclusions**: RVT is high in patients with CAT. The presence of RVT at 6 months was associated with an increased risk of recurrent VTE over 5 years.

## Simple Summary

This research aims to address the ongoing debate regarding the role of residual venous thrombosis (RVT) as a predictor of recurrent venous thromboembolism (VTE) in patients with cancer-associated thrombosis (CAT). This study analysed 511 CAT patients treated with tinzaparin over a follow-up period of nearly 18 months, focusing on the prevalence of RVT and its association with clinical outcomes such as VTE recurrence and bleeding. The findings show that RVT at six months significantly increased the risk of VTE recurrence within five years, especially in patients with metastases. These results suggest that monitoring RVT in CAT patients could inform long-term management strategies to reduce recurrent VTE, providing valuable insights for the research community into improving patient outcomes.

## 1. Introduction

Residual venous thrombosis (RVT) is defined as the long-term persistence of thrombotic material in the veins following a venous thrombotic event [1]. The presence of RVT has been determined in various studies employing different methodologies [2]. Imaging techniques, such as compression or Doppler ultrasonography (US), computed tomography (CT) angiography, and perfusion lung scans, have been used to evaluate RVT [2,3].

Several authors have emphasised the significance of RVT in patients with venous thromboembolism (VTE). Prandoni et al. evaluated the long-term impact of RVT in patients with deep vein thrombosis (DVT), concluding that the presence of RVT doubles the risk of recurrent VTE, post-thrombotic syndrome, arterial thromboembolic events, and cancer [1]. Subsequently, they evaluated the presence of RVT and conducted serial D-dimer measurements for decision making regarding the discontinuation of anticoagulation, finding that patients with early venous recanalisation have a lower risk of recurrent VTE [4].

However, information regarding RVT is limited in patients with cancer-associated thrombosis (CAT). Napolitano et al. conducted a prospective study in patients with lower limb DVT and cancer and found that 69.7% of the patients had RVT at six months. Moreover, patients without RVT had a significantly lower risk of VTE recurrence compared to those with RVT after discontinuing anticoagulation for six months [5]. The identification of predictive variables of VTE recurrence has the potential to guide strategies for secondary prevention and identify patients in whom anticoagulation should be extended [2].

The aim of this study was to assess the presence of RVT in patients with CAT treated with tinzaparin and to identify variables associated with RVT.

## 2. Materials and Methods

### 2.1. Design

We conducted a retrospective, single-centre cohort study of consecutive patients attended to at the Virgen del Rocio University Hospital in Seville (Spain) from December 2007 to July 2022. This study was approved by the Ethics Committee of the centre according to Spanish Regulatory Authorities (0511-N-22) and conducted in accordance with the principles of the Declaration of Helsinki. All study documents were prepared in accordance with the Good Clinical Practice guidelines (CPMP/ICH/135/95). Individual data were intentionally obtained for this study, anonymised, and protected in accordance with the European Union Directive 2016/679 of the European Parliament and the European Council, dated 27 April 2016.

### 2.2. Patient Selection

Patients over the age of 18 diagnosed with VTE and active cancer were selected for this study. VTE included pulmonary embolism (PE), DVT of the lower or upper limbs, and thrombosis at unusual locations. Unusual location VTE was defined as thrombosis affecting any venous region other than PE or DVT of the lower limbs.

The diagnosis of cancer was histologically confirmed, and active cancer was defined as receiving oncological treatment, in progression, metastatic, or recently diagnosed within six months before the VTE event. All patients underwent confirmatory imaging of the thrombotic event, which was performed using CT, lung scintigraphy, Doppler, or compressive US. In patients where residual venous thrombosis was assessed by CT scan, we reviewed the CT scans performed in the context of cancer follow-up. In those patients with lower limb DVT, the evaluation of residual venous thrombosis was conducted through compressive ultrasound. Residual venous obstruction in the CT scan was defined as when thrombosis was identified by the radiologist in the follow-up CT scan. For the US investigation, RVT was defined as a vein transverse diameter greater than 2 mm.

Follow-up data were updated for all patients until November 2022, except for patients who had died, for whom the date of death was registered as the last day of follow-up. Digital reports from the VTE unit, oncology, internal medicine, emergency, and primary care were used to update patient follow-up. This approach enabled the collection of information about clinically relevant bleeding (CRB) events, recurrent VTE, anticoagulant treatment, RVT, and mortality.

### 2.3. Outcomes

The primary outcome was to determine the percentage of CAT patients with RVT at 6 months and throughout the entire follow-up period. The secondary outcomes were to (a) identify variables associated with RVT at 6 months and (b) determine if RVT at 6 months is a predictor of CRB or of recurrence of VTE within 5 years.

CRB was defined as the combination of major bleeding and clinically relevant non-major bleeding. According to the criteria set by the International Society of Thrombosis and Haemostasis [6], major bleeding was characterised by any of the following: (1) resulting in death; (2) occurring in a critical area or organ (such as intracranial, intraocular, intraspinal, intraarticular, pericardial, retroperitoneal, or intramuscular with compartment syndrome); (3) resulting in a decrease of more than 2 g/dL of haemoglobin; or (4) requiring the administration of at least 2 units of red blood cells. Clinically relevant non-major bleeding was defined as bleeding leading a patient to seek care in a hospital or outpatient emergency department or requiring medical evaluation. Recurrent DVT was defined as a new non-compressible vein segment, or an increase of at least 4 mm in vein diameter compared to the previous measurement on venous ultrasound. Recurrent pulmonary embolism (PE) was defined by the presence of a new ventilation–perfusion mismatch on a lung scan or a new intraluminal filling defect on chest spiral CT in patients experiencing acute respiratory symptoms. Recurrence of VTE, such as thrombosis in other locations, required confirmatory testing through CT or US. All events were reviewed by two researchers (CRL and VGG). In case of disparity in the interpretation, a third investigator (LJP) reviewed the case to ensure adequate adjudication of the event.

### 2.4. Statistical Analysis

Continuous variables were reported as mean with standard deviation (SD), or as median with interquartile range (percentile 25–75) when appropriate. Categorical variables were presented as counts and percentages. Standardised mean differences were calculated using the d value, where differences greater than 20% were considered clinically significant [7,8]. This value is expressed as a percentage to simplify interpretation and comparison across different studies or measurements. By standardising the difference, the effect of the units is eliminated, enabling a more consistent interpretation of the effect size.

Curve comparisons were conducted using the log-rank (Mantel–Cox) test, along with Cox regression with non-parametric adjustment for proportional hazards for the incidence of RVT, VTE recurrences, CRB, and death. Due to the association found between RVT at 6 months and recurrent VTE within 5 years, we conducted a competing risk analysis and univariate and multivariate analyses with clinically relevant variables that could be associated with recurrent VTE. The competing risk analysis, as determined using the Fine–Gray model [9,10], was performed to assess how deaths affected the ability to observe VTE recurrences. The Cox regression analysis, both univariable and multivariable, was conducted, incorporating the following variables: Eastern Cooperative Oncology Group (ECOG) performance status (0–1 vs. >1), metastasis (yes vs. no), sex (male vs. female), age (<65 years vs. ≥65 years), stop anticoagulant treatment (yes vs. no), VTE location (PE vs. DVT), and RVT at 6 months (yes vs. no). The subgroup analysis also included the variables that were significant in the comparative analysis of both groups. Following this, multivariate analysis was performed. Statistical significance was set at a *p*-value < 0.05. The analyses were conducted using SPSS version 29, OpenEpi (http://www.openepi.com/Menu/OE_Menu.htm; accessed on 7 June 2024), and R software. The specific R libraries employed for this analysis included library (tidycmprsk), library (survival), library (ggsurvfit), library (gtsummary), library (ggplot2), and library (condSURV). For R software, the version used was RStudio Team (2021), and the source URL is http://www.rstudio.com/; accessed on 7 June 2024.

## 3. Results

### 3.1. Study Population

A total of 511 patients with CAT receiving anticoagulant treatment with tinzaparin and at least one follow-up CT were included in the analysis. The mean age was 64.1 years ± 13.4 years (mean ± SD), with a slight predominance of males (53.5% vs. 46.4%). The median period between cancer diagnosis and the occurrence of VTE was 7 months (p25–p75: 3.3–26). The median duration of anticoagulant therapy was 9.4 months (p25–p75: 5.9–17.8). Regarding the location of the index VTE, 38.3% had PE, 35.6% DVT, 16.4% PE plus DVT, and 9.5% VTE in an unusual location (Table 1).

### 3.2. Primary Outcome

**Figure 1:**
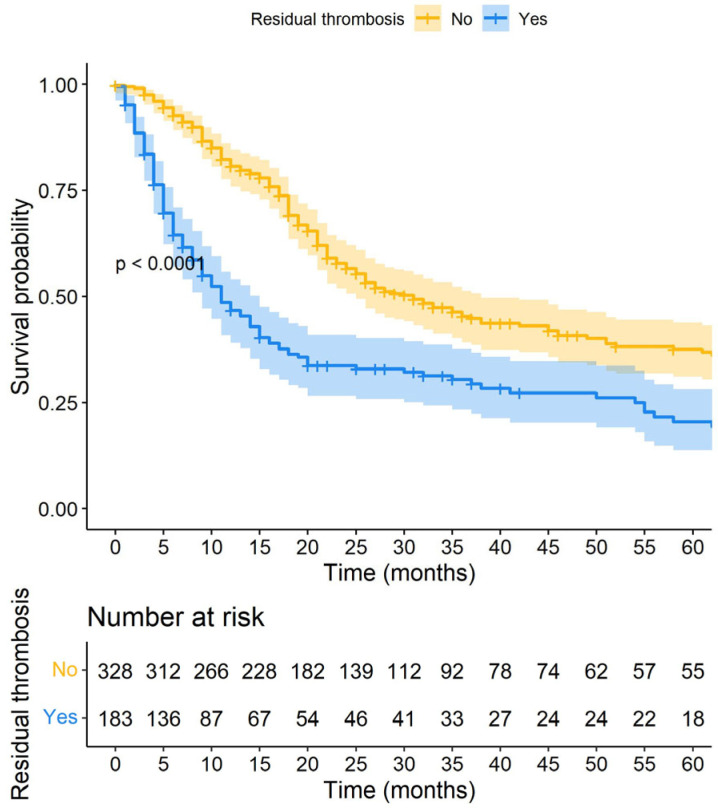
Survival of patients with cancer-associated thrombosis according to residual vein thrombosis.

At 6 months, 55.5% of the patients had RVT and 16% had died (n = 82). Over a median follow-up of 17.6 months (p25–75: 7.9–34), 183 of 511 (35.8%) presented RVT. The median time to resolution of thrombosis was 4.5 months (p25–p75: 2.9–8.6). The neoplasms most associated with RVT were colorectal (15.3%), gynaecological (13.6%), breast (11.4%), and lung (10.9%). Conversely, those least commonly associated with RVT were prostate (4.3%), gastric (2.1%), head and neck cancer (1.6%), and brain (1.6%). During the follow-up period, 319 patients (62.4%) died. Patients with persistent RVT exhibited significantly worse survival outcomes (hazard ratio [HR] 1.79, 95% confidence interval [CI] 1.43–2.25) (Figure 1).

### 3.3. Secondary Outcomes

In the univariate analysis, the variables associated with RVT were as follows: incidental VTE (45.3% vs. 26.3%; HR: 0.72, 95% CI 0.53–1; *p* = 0.047), neoplasm location (pancreas or gynaecological vs. others) (55.6% vs. 32.6%; HR 1.63; 95% CI 1.15–2.32; *p* = 0.006), ECOG performance status > 1 (56.9% vs. 34.2%; HR 2.7; 95% CI 1.82–4.02; *p* < 0.001), and the presence of metastasis (41% vs. 30.2%; HR 2.1; 95% CI 1.56–2.83; *p* < 0.001) (Table 2). In the multivariate analysis, the variables associated with RVT were as follows: ECOG performance status > 1 (HR 2.47; 95% CI 1.65–3.7), presence of metastasis (HR 1.88; 95% CI 1.39–2.56), and cancer location (pancreas or gynaecological vs. others) (HR 1.6; 95% CI 1.13–2.28) (Table 2).

**Figure 2:**
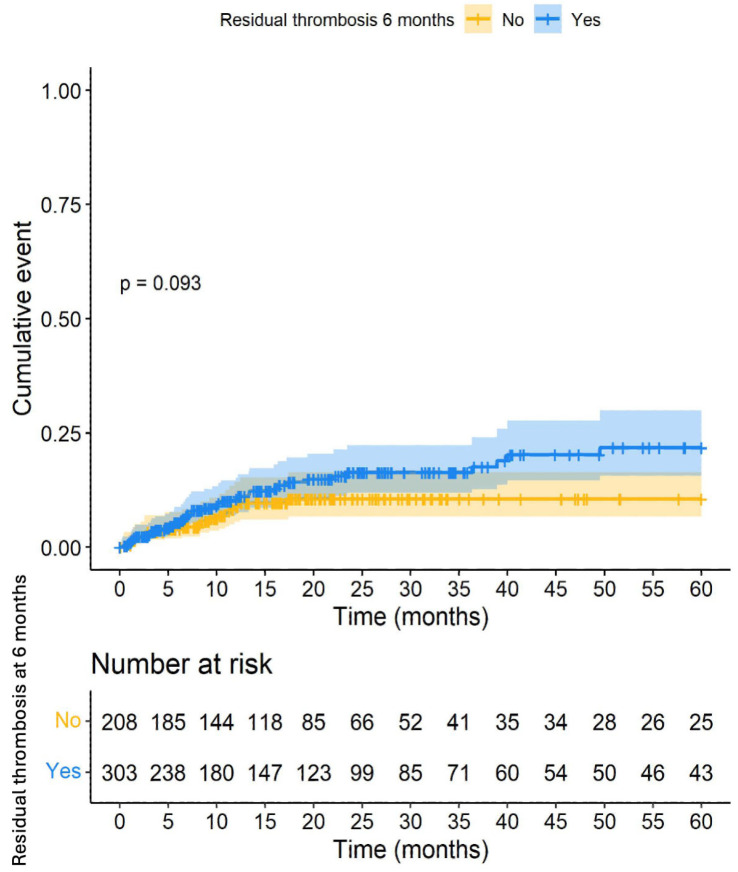
Clinically relevant bleeding within 5 years according to the presence of residual vein thrombosis at 6 months.

Within 5 years, there were 57 CRB events (11.2%; 95% CI: 8.6–14.2). No differences were found when analysing whether RVT at 6 months was associated with a higher risk of CRB at 5 years (Figure 2).

**Figure 3:**
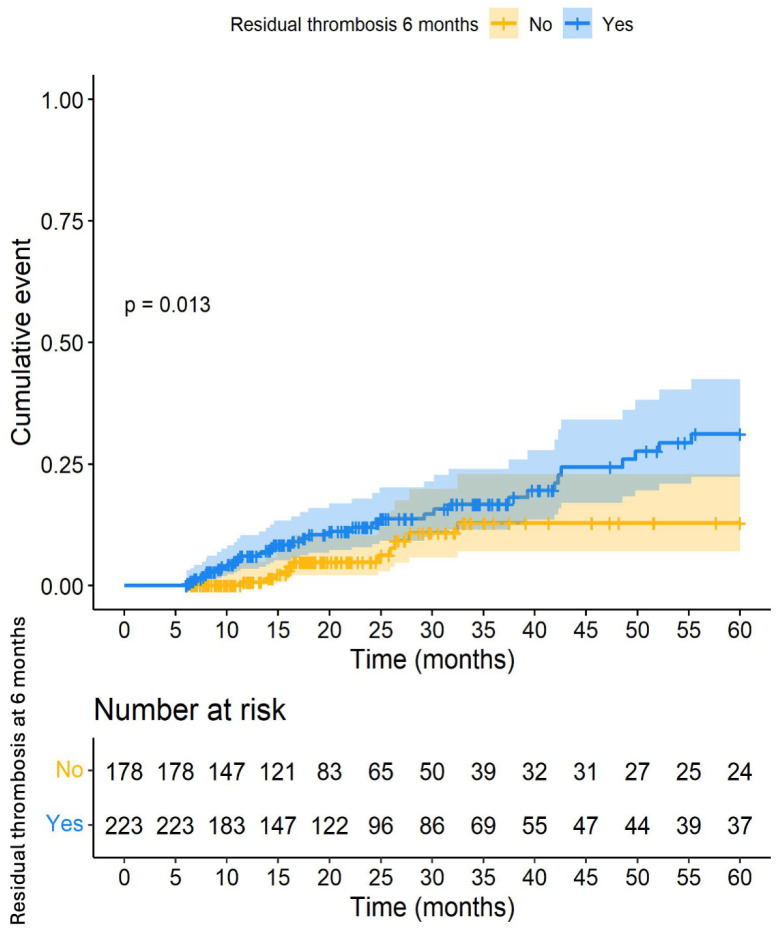
Recurrence of venous thromboembolism within 5 years according to the presence of residual vein thrombosis at 6 months.

Within 5 years, there were 67 VTE recurrences (13.1%, 95% CI: 10.3–16.4). Anticoagulant treatment was discontinued in 189 patients, with a follow-up after discontinuation of 24.1 months (p25–p75: 11.8–57.7). It was observed that patients with RVT at 6 months had a higher risk of VTE recurrence within 5 years (Figure 3). We conducted a competing risk analysis, which continued to show that RVT at 6 months was associated with VTE recurrence within 5 years (sub-hazard ratio [sHR]: 2.14; 95% CI: 1.24–3.69; *p* = 0.006). Multivariate analysis of the variables associated with VTE recurrence identified the presence of metastases (HR: 1.7; 95% CI: 1.1–2.9) and RVT at 6 months (HR: 2.1; 95% CI: 1.2–3.7) (Table 3).

## 4. Discussion

Our data suggest that in patients with CAT, RVT was present in 55.5% at 6 months and throughout the follow-up period in 35.8%. The presence of RVT at 6 months was associated with a higher risk of recurrence over 5 years. Additionally, the heterogeneity of thrombosis resolution was found to depend on the type of cancer. Moreover, the presence of metastasis and RVT was associated with recurrent VTE.

The prevalence of RVT in CAT patients is not well established due to the lack of consensus on the definition of RVT, which has led to methodological differences in the studies available, preventing valid comparisons [2]. While RVT has been widely studied, data specific to cancer patients are scarce. In our cohort, the prevalence of RVT determined by CT was 59.3% (n = 303) at 6 months, being 35.8% (n = 181) at 17 months. Dentali et al. reported a prevalence of 45.8% at 3 months [11], but this group only analysed RVT in the lower limbs using compression US, whereas the present study included VTE in different locations.

We observed that the risk of RVT is higher in some cancer locations (pancreatic and gynaecological), in patients with ECOG performance status > 1, and in those with metastases. The association of these variables with RVT may indicate the aggressiveness of the cancer or an advanced oncological condition [12,13].

As other authors have described previously, it was expected to find that the presence of metastasis is associated with recurrent VTE [14,15,16]. The existence of RVT may be indicative of a hypercoagulable state and inflammation [1], which may be associated with post-thrombotic syndrome [17] and higher mortality [11]. Furthermore, it has been suggested that the formation of thrombi creates a microenvironment conducive to cancer growth and dissemination [18]. On the other hand, the increased risk of VTE recurrence associated with RVT has been analysed previously by other authors, with controversial or negative results [11,19]. In a cohort of patients with first unprovoked DVT, Cosmi et al. found no association between RVT and recurrent VTE [19]. However, in a cohort of CAT patients with isolated distal DVT, Dentali et al. reported that patients with RVT seemed to have a higher risk of presenting recurrent events (odds ratio 2.30; 95%CI 0.73–7.22) [11]. On the other hand, Napolitano found that in patients with CAT in whom anticoagulant treatment was discontinued, the presence of RVT in patients with lower limb DVT was associated with a higher risk of recurrent VTE [5]. Likewise, it could be suggested that RVT may indicate elevated procoagulant activity even during anticoagulation, although this hypothesis should be evaluated and verified in future studies. On the other hand, through Cox regression analysis, we did not find an association between anticoagulation discontinuation and recurrent VTE, although other studies have documented this association [20,21]. This could be due to several factors: (1) the duration of anticoagulant therapy was prolonged; (2) the exposure time after anticoagulation discontinuation over the 5 years was not sufficient; and (3) lack of study power.

This study has several strengths. Firstly, the patients enrolled in this study were derived from consecutive patients evaluated in a VTE unit, which implies analysis of real-world data with better extrapolation or external validity of the results compared to clinical trials, in which patients are selected. Indeed, many of the patients included in our cohort would not have been able to participate in clinical trials due to not meeting the inclusion criteria (i.e., bad performance status and short life expectancy). Secondly, there is a paucity of scientific evidence on residual thrombosis in cancer patients. This study provides a sufficiently large patient cohort for the analysis to support the association of RVT with the variables identified. These findings may provide support for other authors intending to conduct research in this area.

Nevertheless, it is crucial to recognise the inherent limitations of our study. First, it must be noted that this study was based on observational data rather than controlled conditions. Consequently, it is possible that unidentified factors between the two groups may have influenced the outcomes. Second, the results of this study were derived from a single hospital centre, which could have an impact on the applicability of the findings to other healthcare systems that employ different protocols for the management of CAT patients.

For years, studies have been carried out to identify and validate variables or factors associated with bleeding or VTE recurrence in patients with CAT [14,15,16,22,23,24,25,26,27,28,29,30,31]. The problem often stems from the cohort analysed and the variables ultimately included in the predictor model for the complication. A recently published study identified risk factors for bleeding and VTE recurrence in patients with CAT [20]. However, one of the limitations of that study was that the patients were from a clinical trial, thereby limiting the external validity of the results. Nevertheless, the future of research lies in having high-quality data and integrating real-world data with that obtained from clinical trials.

## 5. Conclusions

The presence of RVT is frequent in patients with CAT at 6 months and during long-term follow-up. The presence of RVT at 6 months was associated with an increased risk of recurrent VTE over 5 years. The identification of variables associated with RVT, as well as the association found between RVT and VTE recurrence, may help to determine the intensity of anticoagulation required in patients with CAT.

## Figures and Tables

**Table 1:** Demographic and clinical characteristics of patients according to the presence or absence of residual venous thrombosis.

VARIABLE	Residual Thrombus No (n = 328)	Residual Thrombus Yes (n = 183)	Total Cohort (n = 511)	d Value
**Age (years), mean ± SD, (n = 511)**	62.6 (13)	64.1 (13.4)	63.1 (13.2)	11.4%
**Sex, n (%), (n = 511)**				4.1%
Female	160 (48.7)	85 (46.4)	245 (47.9)	
Male	168 (51.2)	98 (53.5)	266 (52)	
**Incidental VTE, n (%), (n = 511)**	188 (57.3)	67 (36.6)	255 (49.9)	−34.5%
**VTE event, n (%), (n = 511)**				
DVT	74 (22.5)	108 (59)	182 (35.6)	67.2%
PE	152 (46.3)	44 (24)	196 (38.3)	−38.3%
PE + DVT	67 (20.4)	17 (9.2)	84 (16.4)	−24.8%
Unusual VTE	35 (10.6)	14 (7.6)	49 (9.5)	−8.3%
**Cancer type, n (%), (n = 511)**				
Brain	4 (1.2)	3 (1.6)	7 (1.3)	2.8%
Colorectal	71 (21.6)	28 (15.3)	99 (19.3)	−13%
Breast	49 (14.9)	21 (11.4)	70 (13.6)	−8.3%
Lung	48 (14.6)	20 (10.9)	68 (13.3)	−8.9%
Pancreatic	11 (3.3)	15 (8.1)	26 (5)	18.3%
Kidney	17 (5.1)	8 (4.3)	25 (4.8)	−3%
Bladder	16 (4.8)	13 (7.1)	29 (5.6)	8.2%
Prostate	12 (3.6)	8 (4.3)	20 (3.9)	3%
ENT	15 (4.5)	3 (1.6)	18 (3.5)	−12.9%
Gynaecological	21 (6.4)	25 (13.6)	46 (9)	20.9%
Gastric	3 (0.9)	4 (2.1)	7 (1.3)	8.7%
Other	47 (14.3)	25 (13.6)	72 (14)	−1.6%
**ECOG performance status, n (%), (n = 495)**				
ECOG 0	112 (34.1)	56 (30.6)	168 (32.8)	−6.1%
ECOG 1	180 (54.8)	96 (52.4)	276 (54)	−3.9%
ECOG 2	19 (5.7)	23 (12.5)	42 (8.2)	20.6%
ECOG 3	3 (0.9)	5 (2.7)	8 (1.5)	12.1%
ECOG 4	0 (0)	1 (0.5)	1 (0.2)	10.0%
**Metastases, n (%), (n = 510)**	158 (48.1)	110 (60.1)	268 (52.4)	19.7%
**Metastases (single/multiple), n (%), (n = 261)**				
Single	75 (22.8)	68 (37.1)	143 (27.9)	26.4%
Multiple	83 (25.3)	35 (19.1)	118 (23.1)	−12.0%
**Histology, n (%), (n = 511)**				
Adenocarcinoma	152 (46.3)	87 (47.5)	239 (46.7)	2.0%
Epidermoid	34 (10.3)	8 (4.3)	42 (8.2)	−17.9%
Ductal	33 (10)	13 (7.1)	46 (9)	−8.3%
Sarcoma	12 (3.6)	11 (6)	23 (4.5)	9.6%
Urothelial	18 (5.4)	11 (6)	29 (5.6)	2.1%
Glioblastoma	2 (0.6)	3 (1.6)	5 (0.9)	8.5%
Clear cell	11 (3.3)	5 (2.7)	16 (3.1)	−1.5%
Small cell lung	3 (0.9)	3 (1.6)	6 (1.1)	5.4%
Neuroendocrine	7 (2.1)	1 (0.5)	8 (1.5)	−10.5%
Others	43 (13.1)	25 (13.6)	68 (13.3)	1.2%
**Central venous catheter, n (%), (n = 459)**	86 (26.2)	49 (26.7)	135 (26.4)	0.9%
**Oncological treatment, n (%), (n = 509)**	235 (71.6)	132 (72.1)	367 (71.8)	0.9%

Abbreviations: SD: standard deviation; ENT: ear, nose, and throat; ECOG: Eastern Cooperative Oncology Group; VTE: venous thromboembolism; DVT: deep vein thrombosis; PE: pulmonary embolism.

**Table 2:** Univariate and multivariate analyses of risk factors for residual vein thrombosis in patients with cancer and venous thromboembolism.

	Residual Thrombus, n (%)	Univariate Analysis HR (95% CI)	*p*-Value	Multivariate Analysis HR (95% CI)	*p*-Value
**ECOG performance status**		2.7 (1.8–4.0)	<0.001	2.5 (1.7–3.7)	<0.0001
− **0–1 (n = 444)**	152 (34.2)				
− **>1 (n = 51)**	29 (56.9)				
**Histology**			0.49		
− Other histology (n = 457)	154 (33.7)				
− Lymphoma, sarcoma, myeloma, glioblastoma (n = 44)	22 (50.0)				
**Incidental VTE**		0.7 (0.5–1.0)	0.047	1.3 (0.9–1.8)	0.12
− No (n = 256)	116 (45.3)				
− Yes (n = 255)	67 (26.3)				
**Metastasis**		2.1 (1.6–2.8)	<0.001	1.9 (1.4–2.6)	<0.0001
− No (n = 242)	73 (30.2)				
− Yes (n = 268)	110 (41.0)				
**Pulmonary embolism**			0.49		
− No (n = 228)	120 (52.6)				
− Yes (n = 283)	63 (22.3)				
**Lower limb deep vein thrombosis**		1.1 (0.8–1.5)	0.51		
− No (n = 293)	77 (26.3)				
− Yes (n = 218)	106 (48.6)				
**Cancer location**		1.6 (1.2–2.3)	0.006	1.6 (1.1–2.3)	0.009
− Other cancer locations (n = 439)	143 (32.6)				
− Pancreas or gynaecological (n = 72)	40 (55.6)				

Abbreviations: ECOG: Eastern Cooperative Oncology Group; VTE: venous thromboembolism.

**Table 3:** Subgroup analyses in patients with cancer-associated thrombosis and venous thromboembolism recurrence within 5 years.

	VTE Recurrences, n (%)	Univariate COX Regression HR (95% CI)	*p*-Value	Multivariate COX Regression HR (95% CI)	*p*-Value
**Residual vein thrombosis within 6 months**		2.1 (1.2–3.6)	0.011	2.1 (1.2–3.7)	0.011
− Yes (238)	44 (18.5)				
− No (191)	17 (8.9)				
**ECOG performance status**		0.5 (0.1–1.9)	0.2	0.2 (0.03–1.7)	0.15
− 0–1 (n = 444)	61 (13.7)				
− >1 (n = 51)	2 (3.9)				
**Metastasis**		1.6 (0.9–2.6)	0.07	1.7 (1.1–2.9)	0.04
− Yes (n = 268)	34 (12.7)				
− No (n = 243)	33 (13.6)				
**Sex (male vs. female)**		1.1 (0.7–1.8)	0.67	0.9 (0.6–1.7)	0.96
− Male (n = 266)	32 (12.0)				
− Female (n = 245)	35 (14.3)				
**Age (≥65 years vs. <65 years)**		0.9 (0.6–1.5)	0.77	0.8 (0.6–1.6)	0.77
− Older than 65 years (n = 271)	34 (12.5)				
− Younger than 65 years (n = 240)	33 (13.8)				
**Stopped anticoagulant treatment**		0.9 (0.5–1.5)	0.68	1.2 (0.7–2.2)	0.51
− Yes (n = 189)	36 (19%)				
− No (n = 322)	31 (9.6%)				
**VTE location**		1.06 (0.66–1.7)	0.82	1.1 (0.65–1.87)	0.72
− PE (n = 280) *	35 (12.5%)				
− DVT (n = 231)	32 (13.9%)				

Abbreviations: DVT: deep vein thrombosis; ECOG: Eastern Cooperative Oncology Group; VTE: venous thromboembolism; PE: pulmonary embolism. * PE with or without DVT.

## Data Availability

Data are available from the corresponding author upon reasonable request.
